# Fine mapping of *qBK1.2*, a major QTL governing resistance to bakanae disease in rice

**DOI:** 10.3389/fpls.2023.1265176

**Published:** 2023-11-10

**Authors:** Amar Kant Kushwaha, Ranjith Kumar Ellur, Sarvesh Kumar Maurya, Gopala Krishnan S., Bishnu Maya Bashyal, Prolay Kumar Bhowmick, K. K. Vinod, Haritha Bollinedi, Nagendra Kumar Singh, Ashok Kumar Singh

**Affiliations:** ^1^ Division of Crop Improvement and Biotechnology, Indian Council of Agricultural Research (ICAR)-Central Institute for Subtropical Horticulture, Lucknow, India; ^2^ Division of Genetics, Indian Council of Agricultural Research (ICAR)-Indian Agricultural Research Institute, New Delhi, India; ^3^ Division of Plant Pathology, Indian Council of Agricultural Research (ICAR)-Indian Agricultural Research Institute, New Delhi, India; ^4^ National Professor B.P. Pal Chair, Indian Council of Agricultural Research (ICAR)-National Institute of Plant Biotechnology, New Delhi, India

**Keywords:** bakanae, candidate genes, fine-mapping, rice, resistance, SNPs, QTL, NILs (Near isogenic lines)

## Abstract

Bakanae disease caused by *Fusarium fujikuroi* is an emerging disease of rice causing losses in all rice-growing regions around the world. A BC_2_F_2_ population was developed by backcrossing the recurrent parent Pusa Basmati 1121 (PB1121) with the recombinant inbred line RIL28, which harbors a major quantitative trait locus (QTL) governing resistance to bakanae, *qBK1.2*. MassARRAY-based single-nucleotide polymorphism (SNP) assays targeting the genomic region of *qBK1.2* helped in fine mapping the QTL to a region of 130 kb between the SNP markers *rs3164311* and *rs3295562* using 24 recombinants. *In-silico* mining of the fine-mapped region identified 11 putative candidate genes with functions related to defense. The expression analysis identified two significantly differentially expressed genes, that is, *LOC_Os01g06750* and *LOC_Os01g06870*, between the susceptible genotype PB1121 and the resistant genotypes Pusa1342 and R-NIL4. Furthermore, the SNPs identified in *LOC_Os01g06750* produced minor substitutions of amino acids with no major effect on the resistance-related functional motifs. However, *LOC_Os01g06870* had 21 amino acid substitutions, which led to the creation of the leucine-rich repeat (LRR) domain in the resistant genotype Pusa1342, thereby making it a potential candidate underlying the major bakanae-resistant QTL *qBK1.2*. The markers used in the fine mapping program are of immense utility in marker-assisted breeding for bakanae resistance in rice.

## 1 Introduction

Bakanae disease is one of the emerging diseases of rice incited by *Fusarium fujikuroi* (Nirenberg, 1976) (teleomorph: *Gibberella fujikuroi* Sawada (Ito and Kimura, 1931), a filamentous hyphomycete fungus. The disease is prevalent in temperate and tropical rice-growing regions of the world (Amoah et al., 1995; Desjardins et al., 1997; Amatulli et al., 2010; Eğerci et al., 2021). Reported earlier as a minor disease, bakanae has emerged during the last decade as a serious problem in Basmati-growing areas in India, causing yield losses of up to 70% as well as impairing grain quality (Bashyal, 2018). The symptoms of the disease include seedling elongation, seedling mortality, reduced tillers, and elongated non-productive tillers (Bashyal et al., 2016). The disease is primarily seed borne, and secondary spread is through spores on plant parts and soil [Sun, 1975; Anderson and Webster, 2005]. Hot water treatment, and chemical and biological control measures are the major interventions to manage the disease; however, genetic resistance is considered effective (Hayasaka et al., 2001; Hossain et al., 2015; Lee et al., 2018; Sarwar et al., 2018), as it is economical, sustainable, and eco-friendly.

Identification of resistant sources of bakanae disease has found little progress so far due to the complexity of disease responses, which is associated with weather conditions and the pathogen complex known as the *G. fujikuroi* species complex (GFSC) (Desjardins et al., 2000; Wulff et al., 2010; Bashyal and Aggarwal, 2013; Jeon et al., 2013). GFSC is a complex of *Fusarium* species such as *Fusarium andiyazi*, *Fusarium proliferatum*, and *Fusarium verticilloides*, which are also associated with the disease, together with *F. fujikuroi*. This complexity poses challenges in screening rice genotypes under natural conditions. The development of a robust screening technique under artificial inoculation conditions for rice genotypes (Fiyaz et al., 2014) has, however, eased the identification of resistant genotypes. Using this method, several genotypes resistant to bakanae have been identified and successfully utilized in mapping quantitative trait loci (QTLs) governing resistance (Fiyaz et al., 2016). To date, as many as 12 QTLs from various chromosomes have been mapped to impart resistance either through linkage mapping using bi-parental mapping populations or by association mapping using diversity panels comprising germplasm from diverse ecogeographic regions (Yang et al., 2006; Hur et al., 2015; Fiyaz et al., 2016; Volante et al., 2017; Ji et al., 2018; Lee et al., 2018; Chen et al., 2019; Cheon et al., 2019; Kang et al., 2019; Lee et al., 2021; Lee et al., 2022). A maximum number of QTLs have reported on chromosome 1, which includes *qB1*, *qBK1*, *qBK1.1*, *qBK1.2*, *qBK1.3*, *qFfR1*, *qBK1* ^wd^, and *qBK1^z^* (Yang et al., 2006; Hur et al., 2015; Fiyaz et al., 2016; Ji et al., 2018; Lee et al., 2018; Lee et al., 2021). Other QTLs reported are *qBK3.1* on chromosome 3 (Fiyaz et al., 2016), *qBK4_31750955* and *qBK4^T^* on chromosome 4 (Volante et al., 2017; Lee et al., 2022), *qBK_628091* on chromosome 6 (Volante et al., 2017), *qFfrR9* on chromosome 9 (Kang et al., 2019), and *qB10* on chromosome 10 (Yang et al., 2006). However, only one QTL *qBK1* has been fine mapped so far, confined to a 35-kb genomic region, and flanked by InDel markers InDel 18 and InDel 19-14 (Lee et al., 2019). Based on gene expression studies, four putative candidate genes (*LOC_Os01g41770*, *LOC_Os01g41780*, *LOC_Os01g41790*, and *LOC_Os01g41800*) have been identified. The molecular mechanism underlying resistance to bakanae remains largely unknown. However, a transcriptome study has indicated the involvement of jasmonic acid biosynthesis pathway-related genes in the resistant genotype Selenio (Matić et al., 2016).

Since the reported QTLs have been proven effective only against specific isolates of *F. fujikuroi* prevalent in different regions, the wide utilization of the reported QTLs in breeding is limited. However, Lee et al. (2018) found that the pyramiding of the two QTLs *qBK1* and *qBK1^wd^* provided a better level of resistance as compared to individual QTL introgressed lines. Identification of novel QTLs and their deployment into elite cultivars is a proven strategy to broaden the spectrum of resistance and restrict the evolution of pathogens. Furthermore, validating the identified QTLs is of utmost importance prior to their utilization in breeding programs. One of the approaches for validation of a QTL is the development of QTL introgressed near-isogenic lines (QTL-NILs), which can also be used for fine mapping and identification of underlying candidate genes. Also, developing functional markers for the candidate gene bears significance in improving precision in marker-assisted breeding.

In a population generated from the cross between PB1121 and Pusa1342, a major QTL *qBK1.2* was mapped on chromosome 1, which explained 24.74% phenotypic variance (Fiyaz et al., 2016), but remains to be fine mapped. This QTL is effective against the *F. fujikuroi* isolates prevalent in Basmati-growing regions of India. Therefore, this study reports the validation and fine mapping of *qBK1.2* while developing tightly linked markers for their use in marker-assisted breeding.

## 2 Materials and methods

### 2.1 Plant material and development of mapping population

**Figure 1:**
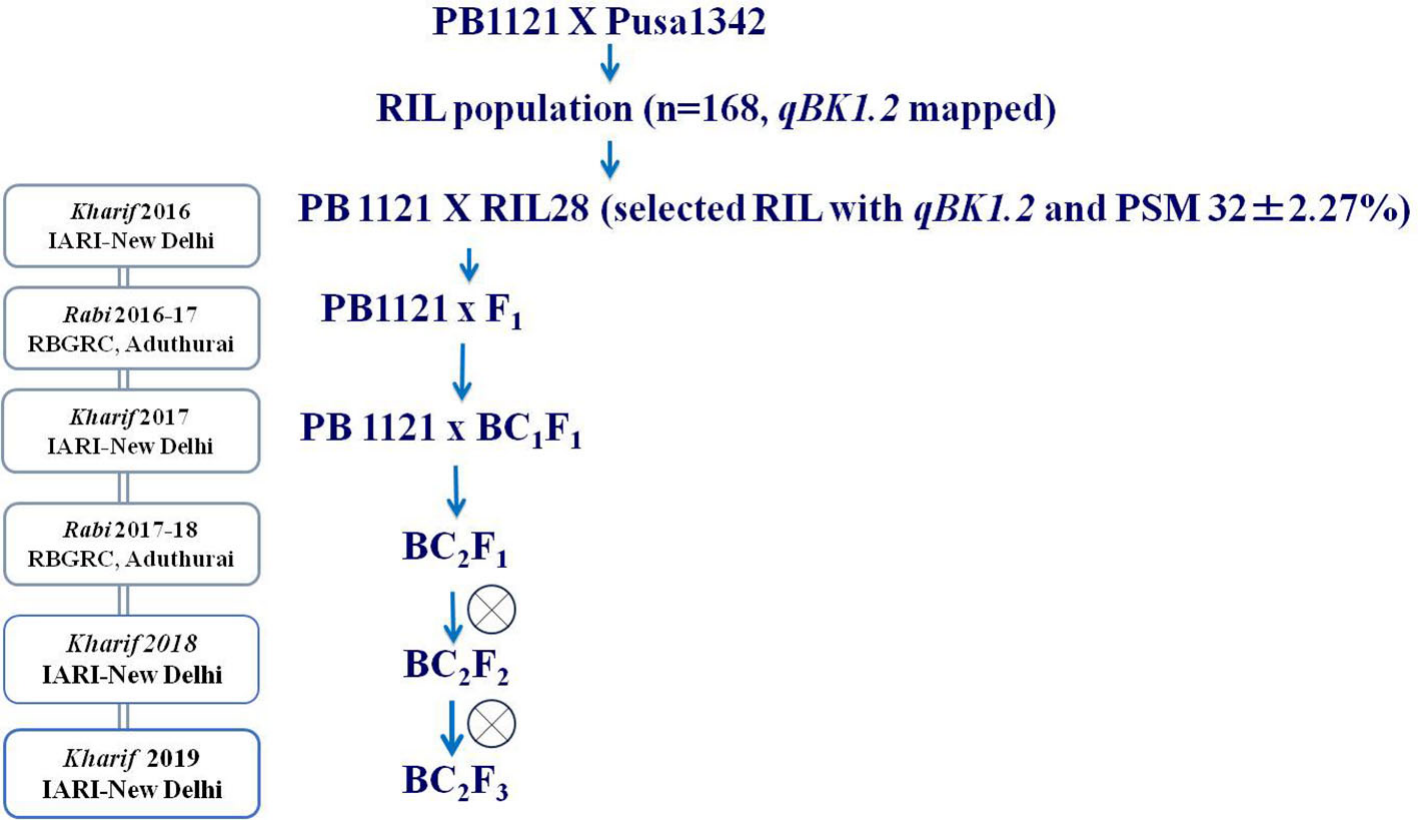
Shuttle breeding scheme for rapid development of backcross mapping population.

RIL28, an individual from a recombinant inbred line (RIL) population (derived from the cross between PB1121 and Pusa1342) carrying the major QTL for bakanae, *qBK1.2*, as confirmed by QTL flanking simple sequence repeat (SSR) markers *RM10153* and *RM5336*, was backcrossed with the susceptible parent PB1121, and a backcross population was generated. The generation advancement was performed by shuttling the material between two locations, i.e., Indian Agricultural Research Institute-New Delhi (IARI-New Delhi) and Rice Breeding and Genetics Research Centre, Tamil Nadu (RBGRC-Tamil Nadu). The F_1_, BC_1_F_1_, and BC_2_F_1_ generations were subjected to foreground selection using the markers *RM10153* and *RM5336* flanking the QTL *qBK1.2*, and background selection was carried out using 28 SSR markers polymorphic between the parental lines (PB1121 and RIL28) to estimate the recurrent parent genome (RPG) recovery. Background recovery was calculated using the formula (1 − (n/N), where n is the number of marker loci heterozygous or homozygous for the recurrent parent allele and N is the total polymorphic markers between parental lines. Genotyping was carried out in the BC_2_F_2_ generation, and phenotyping for bakanae resistance was carried out in the BC_2_F_2:3_ generation. The scheme utilized for developing the BC_2_F_2:3_ population is provided in **Figure 1**.

### 2.2 Phenotypic screening against bakanae disease

**Figure 2:**
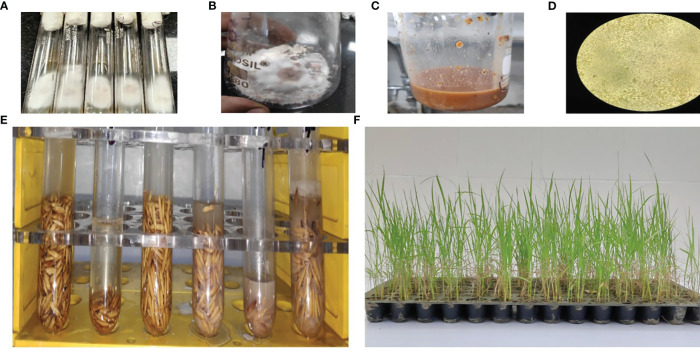
**(A)** Pure culture isolated from diseased plants. **(B)** Autoclaved sorghum seeds were inoculated with pure culture for mass multiplication. **(C)** The suspension was prepared. **(D)** Concentration of the suspension was checked under a light microscope. **(E)** Rice seeds were put in Eppendorf tubes and inoculated with suspension culture for 48 h. **(F)** Sowing and disease scoring was performed in 14 × 7 pot trays.

*F. fujikuroi* isolates were multiplied on sterile sorghum grain at the Division of Plant Pathology, ICAR-Indian Agricultural Research Institute, New Delhi. A 15-day-old culture medium was mashed in sterile water and filtered using a muslin cloth. A suspension culture with a conidial concentration of 1 × 10^6^ was used for inoculation. To screen the rice genotypes, the seeds were soaked in a suspension culture of a virulent *F. fujikuroi* isolate F250 (National Center for Biotechnology Information (NCBI); gene bank accession number KM50526, collected from the northwestern part of India) in the test tubes for 48 hours and incubated at a temperature of 30°C. These seeds were then sown in pot trays (**Figure 2**) and kept in a growth chamber with a day/night temperature of 30°C/25°C and a day/night relative humidity of 60%/80%. Disease scoring on mortality percentage was carried out 12 days after inoculation and classified into different classes (**Table 1**) as described by Fiyaz et al. (2014).

**Table 1:** Phenotypic classification based on percent seedling mortality.

Disease incidence (percent seedling mortality)	Class
0–10	Highly tolerant
11–20	Tolerant
21–40	Moderately tolerant
41–60	Moderately susceptible
61–80	Susceptible
>80	Highly susceptible

### 2.3 Genotypic screening during population development

Fresh leaf samples were collected from 15-day-old seedlings and crushed using a DNA-lyser in Eppendorf tubes. DNA was isolated using the cetyl trimethylammonium bromide (CTAB) protocol described by Murray and Thompson (1980). For the development of the backcross population, foreground selection was carried out using the markers *RM10153* and *RM5336* (**Supplementary Table 1**) flanking the QTL *qBK1.2* (**Supplementary Figures S1** and **S2**). Out of 119 SSR markers polymorphic between PB1121 and Pusa1342, 28 markers were found polymorphic between PB1121 and RIL28 (**Supplementary Table 2**). Parental polymorphism and foreground and background selection were performed in a PCR volume of 10 μl. Each 10-μl reaction volume included 2 μl of template DNA (50 ng), 1 μl of forward primer (5 pm/μl), 1 μl of reverse primer (5 pm/μl), and 3 μl of Taq DNA Polymerase RED 2× master mix (AMPLIQON A/S, Odense, Denmark) and 3 μl of nuclease-free water. PCRs were performed in 96-well PCR plates with a thermal seal in a thermal cycler (Applied Biosystems Veriti™, Foster City, CA, USA). Amplified PCR products were resolved on 3.5% (w/v) agarose gel stained with ethidium bromide. Gels were visualized using a UV-transilluminator gel documentation system (Bio-Rad Gel Doc XR+, Hercules, CA, USA).

### 2.4 SNP genotyping of the recombinants BC_2_F_2:3_ plants

Since there were no polymorphic SSR markers available within the region spanning *RM10153*–*RM5336*, the single-nucleotide polymorphisms (SNPs) polymorphic between PB1121 and Pusa1342 located between the markers, i.e., *RM10153* and *RM5336*, were identified using the re-sequence data available with us (data unpublished). To design the SNP assay, 150-bp upstream and downstream sequences of the SNP were retrieved from the NCBI database using *Oryza sativa* cv. *Nipponbare* as the reference genotype. Then, the SNP assays were designed in assay design suite (ADS) software from Agena^®^ Bioscience (4755 Eastgate Mall, San Diego, CA, USA) using the SNP group file option, and SNP genotyping was performed using MassARRAY^®^, which is based on matrix-assisted laser desorption/ionization–time of flight (MALDI-TOF) mass spectrometry. The steps involved in the genotyping were the amplification of the target region harboring the SNP with PCR following an extension PCR involving primer designed from assay design suit, having the proximal end complementary to the polymorphic base. The resultant mixture from extension PCR was then loaded on SpecroCHIP^®^, which was further placed in a MassARRAY mass spectrometer for signal detection and allele confirmation at the polymorphic site (Ellis and Ong, 2017).

### 2.5 *In silico* search and validation for candidate genes within the fine-mapped region

RNA isolation of the treatment and control was performed 6 days post-inoculation from PB1121, RIL28, and R-NIL4 in three replicates. Total RNA was extracted from shoots using a NucleoSpin RNA kit (Macherey–Nagel, Düren, Germany). Genes were searched within the fine-mapped region using the genome browser in the Rice Genome Annotation Project database (http://rice.uga.edu/) (**Supplementary Table 3**). Primers for real-time PCR analysis of putative candidate genes were designed using the NCBI primer blast tool with a parameter product length of 80–150 bp and Tm of 59°C–61°C. The sequences of these primers are listed in **Table 2**. Rice elF4α (eukaryotic initiation factor-4α) was used as the reference gene for the normalization of expression data. The PCR mixture contained 2.5 μl cDNA (10 times diluted), 5 μl of 2× SYBR green PCR master mix (Bio-Rad, USA), and 100 nM of each gene-specific primer in a final volume of 10 μl. Real-time PCR was performed for all putative candidate genes. Negative template control (NTC) was also performed for each primer pair. Real-time PCR was performed in a Bio-Rad real-time PCR machine (Bio-Rad, USA). All PCRs were performed under the following conditions: 10 min at 95°C and 40 cycles of 15 s at 95°C, 30 s at 60°C and melt curve with a single reaction cycle following conditions 95°C for 15 s, 60°C for 1 min, and dissociation at 95°C for 15 s. Three biological replicates were analyzed for each sample. The relative expression ratio was calculated using the 2^−ΔΔCT^ method (Livak and Schmittgen, 2001).

**Table 2:** List of primers for real-time PCR for validation of candidate genes.

Locus ID	Forward	Reverse
LOC_Os01g06720	ATGGGTTTCTCCGGCAATCT	CTACTCGTCAGCTGAGAAATTTGGG
LOC _Os01g06730	TGCATAGCATTGGTCAGCTC	TGCCATTCGTGAGAGTATGC
LOC _Os01g06750	TTCTGGGGACCGTTCTTGATT	TCAGTGTGCCTGAGAGGTTG
LOC _Os01g06760	CTACAGTTGCCGAGGAAAGC	CCTCACTCAGTGTGCCTGAA
LOC _Os01g06790	GACTTCGGCAGTGGTATGGT	ACTCCATGGAAGTTGTTCTC
LOC _Os01g06836	CAGTGTCCCCTTCTCTTCCA	GCCCCGGTAATTTGGTTACT
LOC _Os01g06870	CAGCCAACAATGACACAACC	AGCTCGCCATGACGATAAGT
LOC _Os01g06876	GCTTCTCTAACTTCTCTTGCTTGG	AGCTGTTGCTAAATGACCCGA
LOC _Os01g06890	GGTATAGGACGCCTCACCAA	ATATCGGCACGATCGTCTTC
LOC _Os01g06900	TTGGTCAAGCGATGTAGCAG	CAGTGAACGGAGTCGTGAGA
LOC _Os01g06920	TGTCGAAAGAATGCAGCAAC	ACCAAAACCCAATCCAAGAA

### 2.6 *In silico* analysis of candidate genes

The sequences of genes, i.e., *LOC_Os01g06750* and *LOC_Os01g06870* of PB1121 and Pusa1324, were generated by incorporating the SNPs, which were identified through re-sequencing of PB1121 and Pusa1342 using Nipponbare as a reference genome (**Supplementary File Alignment**). These sequences were then subjected to SPLIGN analysis to identify if the introns were present (Kapustin et al., 2008). The resulting coding sequences (CDSs) thus generated were then translated, and alignment was performed to identify amino acid substitutions between PB1121 and Pusa1342 (Madeira et al., 2022). Using the translated proteins, prosite analysis was carried out for the identification of differences in defense-related motifs (De Castro et al., 2006).

## 3 Results

**Figure 3:**
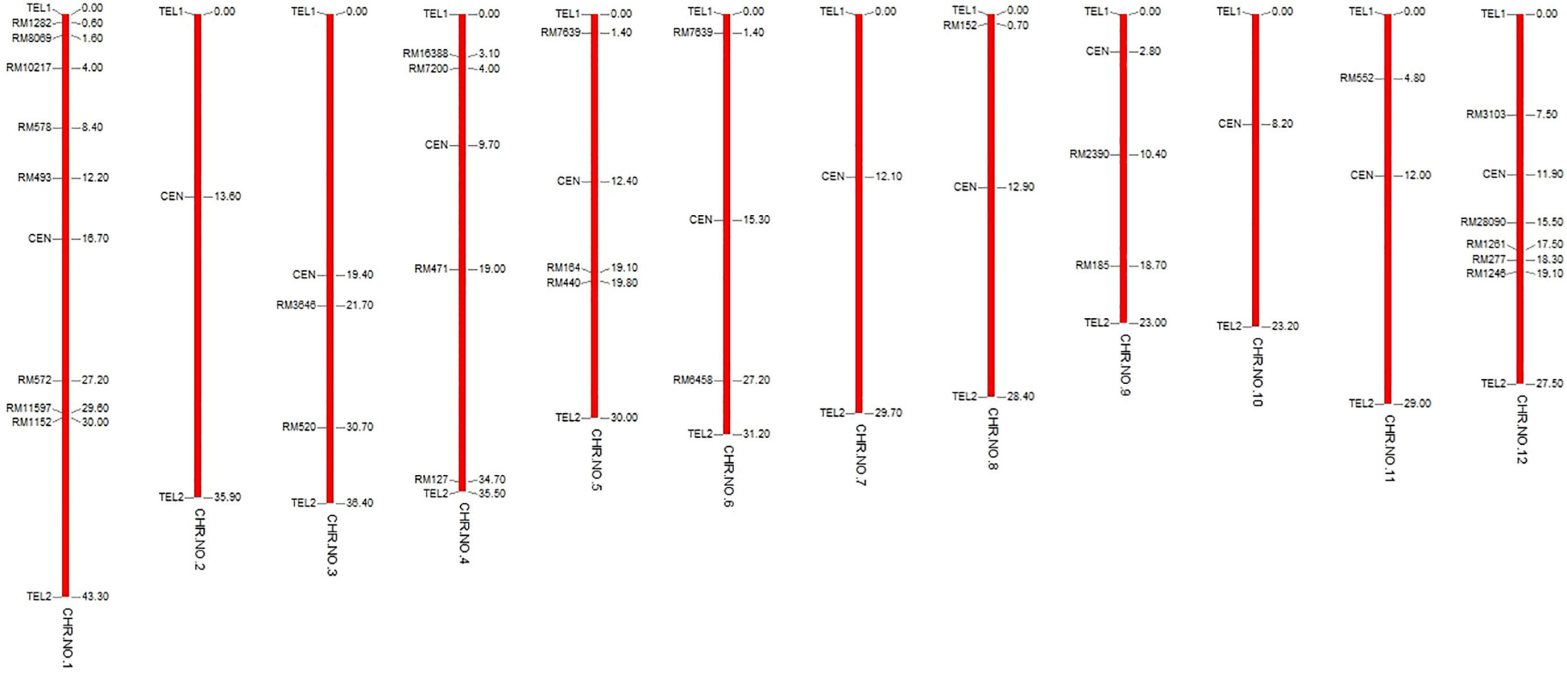
Polymorphism between recurrent parents PB1121 and RIL28.

Among the RILs generated from the cross between PB1121 and Pusa1342, RIL28 was chosen for the study because it carried *qBK1.2* and showed a percent seedling mortality of 32% as compared to 95.33% in the susceptible genotype PB1121 under bakanae infection in the artificial screening conditions (**Figures 2A, B**). Furthermore, RIL28 exhibited 76.47% similarity to PB1121 based on the 119 SSR markers of which 28 were polymorphic (**Figure 3**).

**Figure 4:**
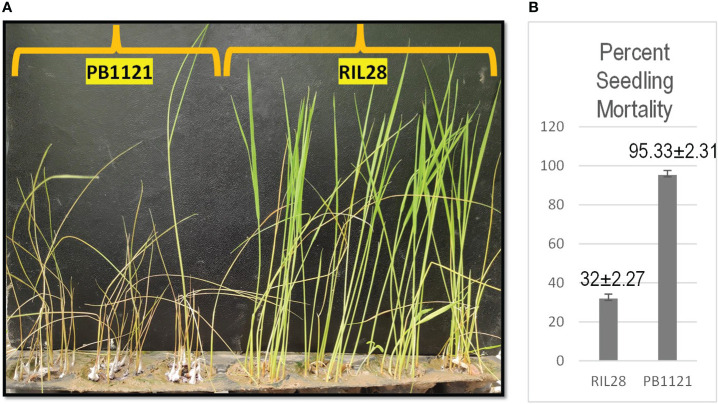
The response of genotypes to *Fusarium fujikuroi* infection. **(A)** Parental lines used for developing population for fine mapping. **(B)** Percent seedling mortality in the parental lines.

The F_1_s from the cross made between PB1121 and RIL28 when subjected to the test of hybridity using the *qBK1.2* linked markers, *RM10153* and *RM5336*, showed one true hybrid out of 12 plants tested. A true F_1_ was backcrossed with PB1121, 35 BC_1_F_1_s were generated with *qBK1.2*, and RPG recovery ranged from 78.57% and 82.14%. Out of these 35 BC_1_F_1_s, an individual plant with RPG recovery of 82.15% was backcrossed with PB1121 to generate 90 BC_2_F_1_s. The RPG recovery among the BC_2_F_1_ plants carrying *qBK1.2* ranged from 85.71% to 96.42%. A BC_2_F_1_ plant with 96.42% RPG recovery was selfed to generate a BC_2_F_2_ population comprising 1,100 individuals. Genotyping with foreground markers followed by phenotyping using *F. fujikuroi* F250 isolate (**Figure 4**) resulted in the identification of 24 BC_2_F_2:3_ recombinants falling in both resistant and susceptible classes.

### 3.1 Disease reaction among the backcross-derived lines

**Figure 5:**
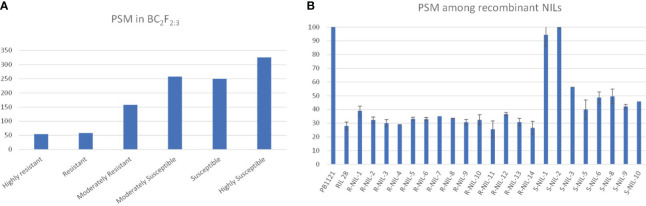
**(A)** Number of classes in different groups based on percent seedling mortality (PSM). **(B)** disease reaction of recombinants.

The backcross population, i.e., BC_2_F_2:3_ families, were phenotyped for bakanae resistance and classified based on disease reaction, i.e., percent seedling mortality. The NILs on artificial inoculation and screening showed significant variation in percent seedling mortality, which ranged from highly susceptible to highly resistant reactions (**Figure 5**).

**Figure 6:**
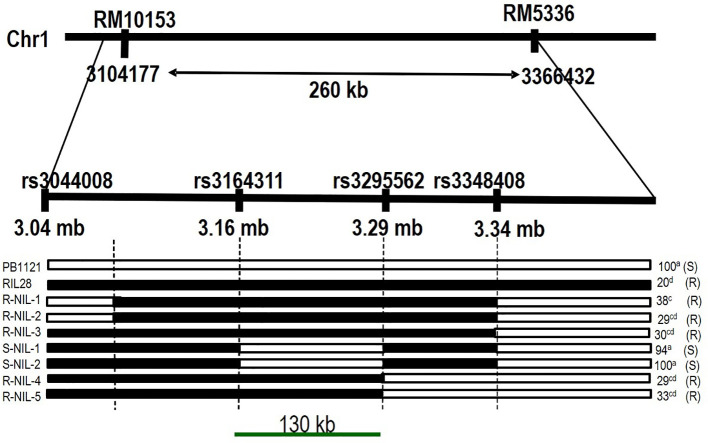
Fine-mapped region of qBK1.2. The QTL region is delimited to 130 kb between SNPs rs3164311 and rs3295562 using recombinants. The values on the right side indicate percent mortality upon inoculation. NIL, near-isogenic line; R-NIL, resistant NIL; S-NIL, susceptible NIL; QTL, quantitative trait locus; SNPs, single-nucleotide polymorphisms.

Based on the marker profile and bakanae screening data of *RM10153* and *RM5336*, a total of 24 recombinants were identified. These recombinants can be categorized into three groups, viz., i) recombinant on the *RM10153* marker side, ii) recombinant on the *RM5336* side, and iii) recombinant on both sides. Four SNPs within the target genomic region were identified based on whole genome re-sequencing data of the parental lines PB1121 and Pusa1342. Genotyping of recombinants with the MassARRAY (MALDI-TOF mass spectrometry)-based SNP genotyping delimited *qBK1.2* to a region of 130 kb between the SNPs *rs3164311* and *rs3295562* (**Figure 6**).

### 3.2 Expression profiling of putative candidate genes

**Figure 7:**
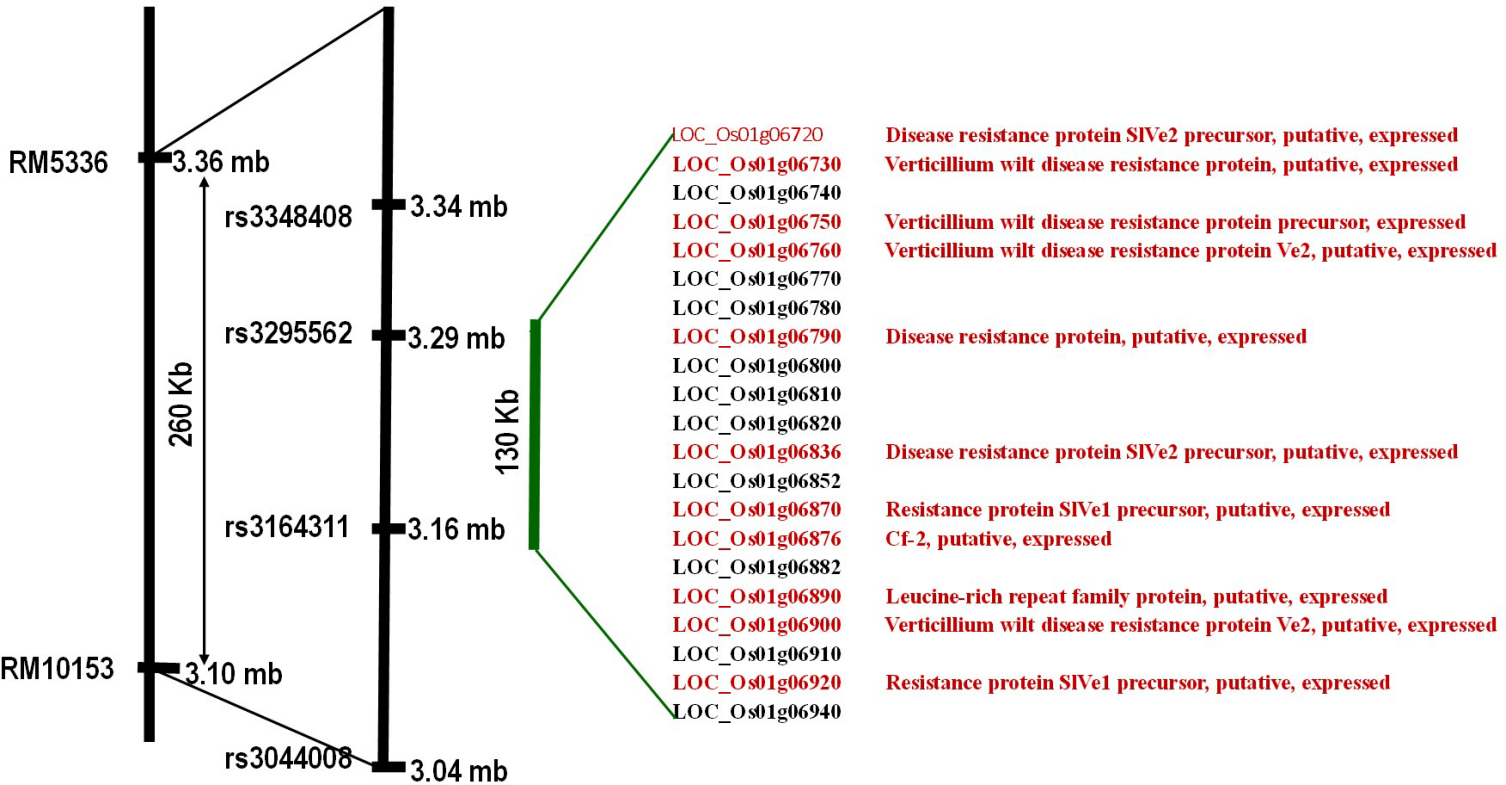
Putative genes underlying the fine-mapped region. Locus IDs in red have a putative function in defense against disease, and IDs in black represent retroposons.

*In-silico* search within the fine-mapped region using a rice genome annotation project database (http://rice.uga.edu/) led to the identification of 21 putative gene models, among which 11 were with annotated disease resistance-related functions (*SlVe1*, *SlVe2*, *Ve1*, *cf-2*, and other *Verticillium* wilt resistance genes) (**Figure 7**).

**Figure 8:**
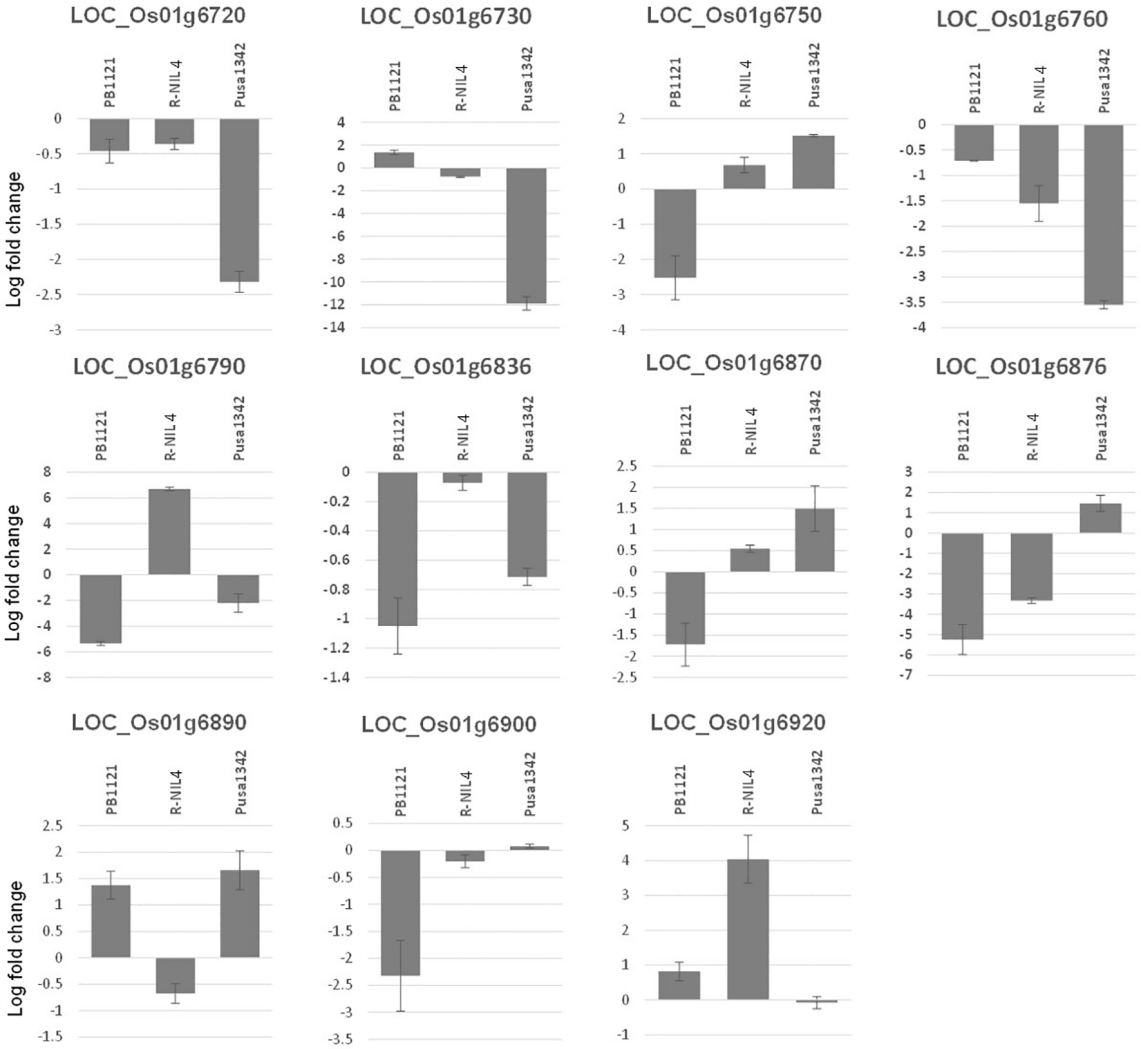
Relative expression (log fold change) of putative candidate genes underlying the fine-mapped region.

Expression of all the 11 putative candidate genes was profiled under mock and inoculated conditions in PB1121, Pusa1342, and Resistant NIL-4 (R-NIL4). Two genes, that is, *LOC_Os01g06750* and *LOC_Os01g06870*, showed significant upregulation when treated with F250 isolate of *F. fujikuroi* (day/night temperature of 30°C/25°C ( ± 3)°C and relative humidity of 60%/8%0 ( ± 10%)) in the resistant genotypes Pusa1342 and R-NIL4, while there was significant downregulation in the susceptible genotype PB1121. Genes such as *LOC_Os01g06720*, *LOC_Os01g06730*, *LOC_Os01g06760*, and *LOC_Os01g06876* had significant differential expression between the resistant genotype Pusa1342 and susceptible genotype PB1121, but not in R-NIL4. The expression of *LOC_Os01g06790* was significantly upregulated in R-NIL4 while downregulated in both Pusa1342 and PB1121 (**Figure 8**). This indicated that genes *LOC_Os01g06750* and *LOC_Os01g06870* could be the potential candidates underlying the major QTL *qBK1.2*. Further analysis indicated that these genes are orthologous to the tomato *Verticillium* wilt resistance gene *Vei*.

### 3.3 *In silico* comparison of candidate genes and products

**Figure 9:**
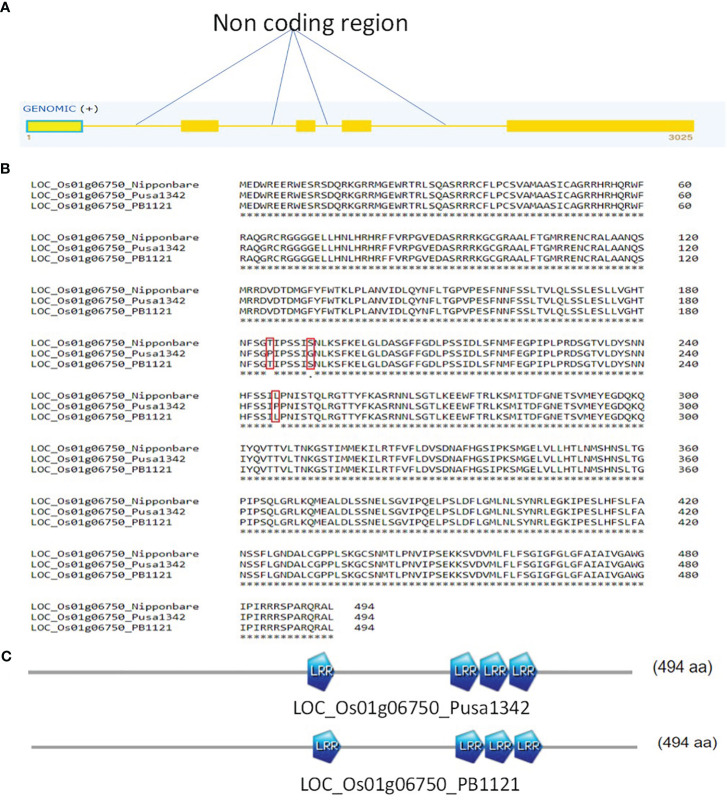
**(A)** Structure of gene *LOC_Os01g06750*; solid yellow boxes represent exons. **(B)** Alignment of proteins from gene *LOC_Os01g06750* from PB1121 and Pusa1342. **(C)** Number of LRR motif in PB1121 and Pusa1342. LRR, leucine-rich repeat.

*LOC_Os01g06750* was predicted to possess five exons (**Figure 9A**). Alignment of the sequences of PB1121 and Pusa1342 led to the identification of one, six, and four SNPs on exons 1, 4, and 5, respectively. Alignment of 494 amino acid translated sequence of PB1121 and Pusa1342 (**Figure 9B**) resulted in the identification of three amino acid substitutions: i) substitution of threonine with proline at 185th amino acid, ii) serein with glycine at 191st amino acid, and iii) leucine with proline at 246th amino acid position. Since these substitutions did not lead to any structural differences (**Figure 9C**), the role of this gene at protein level cannot be confirmed.

**Figure 10:**
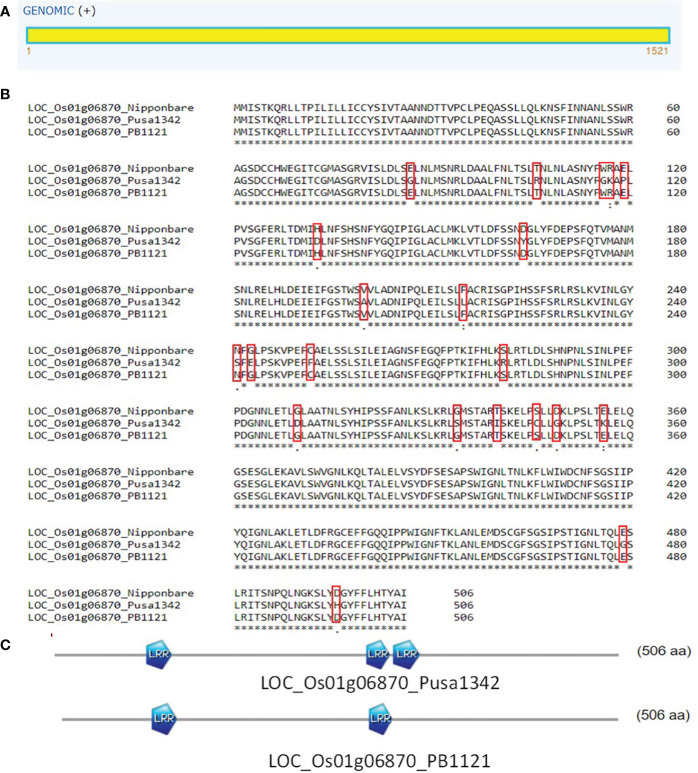
**(A)** Structure of gene *LOC_Os01g06870* represents single exons. **(B)** Alignment of proteins from gene *LOC_Os01g06870* from PB1121 and Pusa1342. **(C)** Reduced number of LRR domains in PB1121 as compared to Pusa1342. LRR, leucine-rich repeat.

*LOC_Os01g06870* was predicted to be an intron-less gene that codes for resistance protein SlVe1 precursor with two leucine-rich repeat (LRR) domains. DNA sequence alignment led to the identification of 30 SNPs between PB1121 and Pusa1342. Translated product alignment resulted in the identification of 21 amino acid substitutions. This led to the creation of an extra LRR domain in the resistant parent Pusa1342 as compared to the susceptible genotype PB1121 (**Figure 10**). Therefore, *LOC_Os01g06870* may be considered as the potential candidate underlying *qBK1.2* conferring tolerance to bakanae disease.

## 4 Discussion

The severity of bakanae disease has been increasing over the years in the Indo-Gangetic plains of India wherein Basmati rice is one of the major crops. The majority of the Basmati rice varieties show susceptibility to bakanae. Although genetic resistance to bakanae is reported by several studies, the sources of resistance lie outside the Basmati gene pool. Naturally, Basmati rice has a narrow genetic base, and hence, the deployment of resistance genes is sought from non-Basmati sources. This necessitates a long breeding process to safeguard the specific Basmati traits along with the recruited trait. Recently, it was established that marker-assisted backcross breeding can ease this cumbersome breeding process and accelerate varietal development with enormous success (Lee et al., 2018).

The QTL qBK1.2 identified on chromosome 1 lies over a span of 260 kb on the short arm flanked by the SSR markers, i.e., RM10153 and RM5336 (Fiyaz et al., 2016). In the current study, we used the approach of fine mapping qBK1.2 using QTL-NILs. NILs provide several advantages such as low background noise and genetic similarity to the sensitive parent. Because of these benefits, NILs are considered ideal for QTL validation, fine mapping, comparative genomics, marker development, gene expression studies, and even deployment as new cultivars. The BC_2_F_2_ population generated in the current study had an RPG recovery of 97.91%. Moreover, the F_2:3_ population provided an opportunity for evaluating resistance reactions under the artificial screening system. The variation observed among the NILs was quantitative and could be due to environmental influence. The environmental influence of disease response makes the management of phenotyping cumbersome, expensive, and time-consuming.

The SNP genotyping among the recombinants delimited qBK1.2 to a region of 130 kb between the SNPs rs3164311 and rs3295562. It was found that when this region was absent in the S-NIL1 and S-NIL2, the disease intensity reached above 94% (**Figure 4**). Since the other regions between the flanking markers were similar to the donor, the likelihood of the region being the candidate locus was very high, making us conclude that the QTL resided in this region. A similar strategy was used to fine map several QTLs in rice including qBK1 (Lee et al., 2019). The fine-mapped region comprised 11 annotated genes with putative disease resistance function. The expression pattern of a gene under mock and challenged inoculation conditions provides significant contrast in its involvement in response under infection (Matić et al., 2016; Cheng et al., 2020). Genes LOC_Os01g41770 and LOC_Os01g41780 were annotated to encode leucine-rich repeat motifs, which were identified to negatively regulate bakanae disease resistance (Lee et al., 2019). Eight of 11 genes that were subjected to expression profiling between the parental lines PB1121 and Pusa1342 showed significant differential expression. The observed differences may be attributed to the response to infection dynamics and/or due to inherent differences in the genomes of these genotypes. Therefore, comparing the expression between the NILs could help identify the genes responding to the infection. Accordingly, the R-NIL4 carrying qBK1.2 in the genetic background of PB1121 was profiled along with the parental lines. The expression pattern ruled out the involvement of five loci, i.e., LOC_Os01g06720, LOC_Os01g06730, LOC_Os01g06760, LOC_Os01g06876, LOC_Os01g06900, and LOC_Os01g06790, in the manifestation of resistance to bakanae because they failed to show a differential response between qBK1.2 carriers (R-NIL4 and Pusa1342) and the non-carrier (PB1121). Two genes, that is, LOC_Os01g06750 and LOC_Os01g06870, have shown significant differential expression between susceptible (PB1121) and resistant individuals (Pusa1342 and R-NIL4). A comparative analysis of encoded protein using BLASTp showed that these genes are orthologues of Verticillium wilt (Verticillium dahliae) resistance gene of tomato, i.e., Ve1, which is rich in leucine-rich repeats. Ve1 gene of tomato was identified to provide resistance in tomato against race 1 strains of V. dahliae and Verticillium albo-atrum (De Jonge et al., 2012). It was also shown that Ve1 requires the cascade of signaling genes, such as Enhanced Disease Susceptibility 1 (EDS1), Non-race-specific Disease Resistance 1 (NDR1), NB-LRR Protein Required for HR-Associated Cell Death 1 (NRC1), ACIF, MAPK/ERK kinase 2 (MEK2), and SERK3/BAK1 (Fradin et al., 2009). The other gene, i.e., Ve2, has been shown to act antagonistically to Ve1 using RNAi, and it was found that Ve1 regulates the expression of defense-related genes by minimizing the effects of Ve2 on these genes (Nazar et al., 2018).

LOC_Os01g06750 is an interrupted gene with four introns, while LOC_Os01g06870 is an uninterrupted gene. The gene sequence variation among the genotypes determines the target phenotype. Although SNPs were identified in LOC_Os01g06750 between PB1121 and Pusa1342, the translated product revealed minor substitution of amino acids with no major effect on functional motifs, i.e., leucine-rich repeat having a role in the resistance-related functions (McHale et al., 2006). This indicates its function in providing tolerance to bakanae at the transcriptional or posttranscriptional level or by generating protein products through alternate splicing of the exons. However, the translated product of LOC_Os01g06870 had 21 amino acid substitutions, which led to the creation of the LRR domain in the resistant individual Pusa1342. The differential expression of LOC_Os01g06870 and functional difference in gene product make it the potential candidate gene in providing tolerance against bakanae disease and can be conclusively proved with functional validation by generating transgenic events in the susceptible genotype.

We conclude that *LOC_Os01g06870* is a potential putative candidate gene conferring tolerance to bakanae disease, underlying the major QTL *qBK1.2* between the SNPs *rs3164311* and *rs3295562*. These markers are resources of immense importance for a rice breeding program targeting to introgress *qBK1.2* for developing bakanae resistance in rice.

## Data availability statement

The raw data supporting the conclusions of this article will be made available by the authors, without undue reservation.

## Author contributions

AK: Data curation, Formal Analysis, Investigation, Validation, Visualization, Writing – original draft. RE: Formal Analysis, Methodology, Writing – review & editing. GK: Conceptualization, Funding acquisition, Project administration, Supervision, Writing – review & editing. SM: Data curation, Writing – review & editing. BB: Resources, Writing – review & editing. PB: Writing – review & editing. HB: Writing – review & editing. KV: Writing – review & editing. NS: Resources, Writing – review & editing. AS: Conceptualization, Project administration, Supervision, Writing – review & editing.
