# Supplementary material for: Fine mapping of qBK1.2, a major QTL governing resistance to bakanae disease in rice
Source: Front Plant Sci. 2023 Nov 10;14:1265176. doi: 10.3389/fpls.2023.1265176 (PMC10667430; doi:10.3389/fpls.2023.1265176)
Supplement: Supplementary file 1 [file DataSheet_1.docx]

***LOC_Os01g06750***

**LOC_Os01g06750_Nipponbare ATGGAGGACTGGAGGGAGGAGAGGTGGGAGTCGAGGTCGGACCAGCGGAAGGGGAGGAGG 60**

**LOC_Os01g06750_Pusa1342 ATGGAGGACTGGAGGGAGGAGAGGTGGGAGTCGAGGTCGGACCAGCGGAAGGGGAGGAGG 60**

**LOC_Os01g06750_PB1121 ATGGAGGACTGGAGGGAGGAGAGGTGGGAGTCGAGGTCGGACCAGCGGAAGGGGAGGAGG 60**

**LOC_Os01g06750_Nipponbare ATGGGCGAGTGGAGGACGAGGCTGTCGCAGGCCTCGCGGAGGCGTTGCTTCTTGCCATGC 120**

**LOC_Os01g06750_Pusa1342 ATGGGCGAGTGGAGGACGAGGCTGTCGCAGGCCTCGCGGAGGCGTTGCTTCTTGCCATGC 120**

**LOC_Os01g06750_PB1121 ATGGGCGAGTGGAGGACGAGGCTGTCGCAGGCCTCGCGGAGGCGTTGCTTCTTGCCATGC 120**

**LOC_Os01g06750_Nipponbare AGCGTCGCGATGGCGGCCTCCATCTGCGCCGGCCGTCGTCATCGCCATCAGAGATGGTTC 180**

**LOC_Os01g06750_Pusa1342 AGCGTCGCAATGGCGGCCTCCATCTGCGCCGGCCGTCGTCATCGCCATCAGAGATGGTTC 180**

**LOC_Os01g06750_PB1121 AGCGTCGCGATGGCGGCCTCCATCTGCGCCGGCCGTCGTCATCGCCATCAGAGATGGTTC 180**

**LOC_Os01g06750_Nipponbare AGGGCACAGGGCCGTTGCCGTGGCGGCGGCGGCGAGCTGTTGCATAATCTCCATCGCCAT 240**

**LOC_Os01g06750_Pusa1342 AGGGCACAGGGCCGTTGCCGTGGCGGCGGCGGCGAGCTGTTGCATAATCTCCATCGCCAT 240**

**LOC_Os01g06750_PB1121 AGGGCACAGGGCCGTTGCCGTGGCGGCGGCGGCGAGCTGTTGCATAATCTCCATCGCCAT 240**

**LOC_Os01g06750_Nipponbare CGATTCTTCGTGCGGCCGGGGGTAGAGGACGCTTCTCGGCGAAGGAAAGGGTGCGGGCGT 300**

**LOC_Os01g06750_Pusa1342 CGATTCTTCGTGCGGCCGGGGGTAGAGGACGCTTCTCGGCGAAGGAAAGGGTGCGGGCGT 300**

**LOC_Os01g06750_PB1121 CGATTCTTCGTGCGGCCGGGGGTAGAGGACGCTTCTCGGCGAAGGAAAGGGTGCGGGCGT 300**

**LOC_Os01g06750_Nipponbare GCGGCGCTGTTCACCGGAATGCGTCGTGAGAACTGCCGCGCGCTGGCCGCGAATCAATCC 360**

**LOC_Os01g06750_Pusa1342 GCGGCGCTGTTCACCGGAATGCGTCGTGAGAACTGCCGCGCGCTGGCCGCGAATCAATCC 360**

**LOC_Os01g06750_PB1121 GCGGCGCTGTTCACCGGAATGCGTCGTGAGAACTGCCGCGCGCTGGCCGCGAATCAATCC 360**

**LOC_Os01g06750_Nipponbare ATGAGAAGGGATGTGGATACGGATATGGGCTTTTACTTCTGGACGAAATTGCCCCTTGCT 420**

**LOC_Os01g06750_Pusa1342 ATGAGAAGGGATGTGGATACGGATATGGGCTTTTACTTCTGGACGAAATTGCCCCTTGCT 420**

**LOC_Os01g06750_PB1121 ATGAGAAGGGATGTGGATACGGATATGGGCTTTTACTTCTGGACGAAATTGCCCCTTGCT 420**

**LOC_Os01g06750_Nipponbare AATGTGATTGACCTACAATACAACTTTTTGACTGGTCCAGTTCCAGAGTCCTTTAACAAC 480**

**LOC_Os01g06750_Pusa1342 AATGTGATTGACCTACAATACAACTTTTTGACTGGTCCAGTTCCAGAGTCCTTTAACAAC 480**

**LOC_Os01g06750_PB1121 AATGTGATTGACCTACAATACAACTTTTTGACTGGTCCAGTTCCAGAGTCCTTTAACAAC 480**

**LOC_Os01g06750_Nipponbare TTCTCCTCTTTGACTGTTCTCCAGCTCAGTAGCTTGGAGAGTTTGCTTGTTGGTCACACC 540**

**LOC_Os01g06750_Pusa1342 TTCTCCTCTTTGACTGTTCTCCAGCTCAGTAGCTTGGAGAGTTTGCTTGTTGGTCACACC 540**

**LOC_Os01g06750_PB1121 TTCTCCTCTTTGACTGTTCTCCAGCTCAGTAGCTTGGAGAGTTTGCTTGTTGGTCACACC 540**

**LOC_Os01g06750_Nipponbare AACTTCTCTGGTACAATACCAAGTTCCATTAGCAACCTCAAATCTTTTAAAGAGCTGGGC 600**

**LOC_Os01g06750_Pusa1342 AACTTCTCAGGCCCAATACCAAGTTCAATCGGCAACCTCAAATCTTTTAAAGAGCTGGGC 600**

**LOC_Os01g06750_PB1121 AACTTCTCTGGTACAATACCAAGTTCCATTAGCAACCTCAAATCTTTTAAAGAGCTGGGC 600**

**LOC_Os01g06750_Nipponbare CTTGATGCAAGTGGGTTTTTTGGAGACCTGCCATCATCAATAGATCTTAGTTTTAACATG 660**

**LOC_Os01g06750_Pusa1342 CTTGATGCAAGTGGGTTTTTTGGAGACCTGCCATCATCAATAGATCTGAGTTTTAACATG 660**

**LOC_Os01g06750_PB1121 CTTGATGCAAGTGGGTTTTTTGGAGACCTGCCATCATCAATAGATCTTAGTTTTAACATG 660**

**LOC_Os01g06750_Nipponbare TTTGAGGGGCCAATACCTCTACCCAGAGATTCTGGGACCGTTCTTGATTACTCAAACAAT 720**

**LOC_Os01g06750_Pusa1342 TTTGAGGGGCCAATACCTCTACCCAGAGATTCTGGAACCGTGCTTGATTACTCAAACAAT 720**

**LOC_Os01g06750_PB1121 TTTGAGGGGCCAATACCTCTACCCAGAGATTCTGGGACCGTTCTTGATTACTCAAACAAT 720**

**LOC_Os01g06750_Nipponbare CATTTCTCGTCCATACTACCAAATATTTCTACTCAACTCAGAGGTACTACCTATTTCAAG 780**

**LOC_Os01g06750_Pusa1342 CATTTCTCGTCCATACCACCAAATATTTCTACTCAACTCAGAGGTACTACCTATTTCAAG 780**

**LOC_Os01g06750_PB1121 CATTTCTCGTCCATACTACCAAATATTTCTACTCAACTCAGAGGTACTACCTATTTCAAG 780**

**LOC_Os01g06750_Nipponbare GCATCAAGAAACAACCTCTCAGGCACACTGAAAGAAGAATGGTTTACAAGACTAAAGTCT 840**

**LOC_Os01g06750_Pusa1342 GCATCAAGAAACAACCTCTCAGGCACACTGAAAGAAGAATGGTTTACAAGACTAAAGTCT 840**

**LOC_Os01g06750_PB1121 GCATCAAGAAACAACCTCTCAGGCACACTGAAAGAAGAATGGTTTACAAGACTAAAGTCT 840**

**LOC_Os01g06750_Nipponbare ATGATAACTGATTTTGGTAATGAAACATCAGTGATGGAATATGAAGGTGATCAAAAGCAA 900**

**LOC_Os01g06750_Pusa1342 ATGATAACTGATTTTGGTAATGAAACATCAGTGATGGAATATGAAGGTGATCAAAAGCAA 900**

**LOC_Os01g06750_PB1121 ATGATAACTGATTTTGGTAATGAAACATCAGTGATGGAATATGAAGGTGATCAAAAGCAA 900**

**LOC_Os01g06750_Nipponbare ATCTACCAAGTGACCACTGTGCTCACAAACAAAGGGTCTACCATCATGATGGAGAAAATA 960**

**LOC_Os01g06750_Pusa1342 ATCTACCAAGTGACCACTGTGCTCACAAACAAAGGGTCTACCATCATGATGGAGAAAATA 960**

**LOC_Os01g06750_PB1121 ATCTACCAAGTGACCACTGTGCTCACAAACAAAGGGTCTACCATCATGATGGAGAAAATA 960**

**LOC_Os01g06750_Nipponbare CTAAGAACCTTTGTATTCCTTGATGTCTCGGATAACGCATTCCACGGAAGCATCCCTAAA 1020**

**LOC_Os01g06750_Pusa1342 CTAAGAACCTTTGTATTCCTTGATGTCTCGGATAACGCATTCCACGGAAGCATCCCTAAA 1020**

**LOC_Os01g06750_PB1121 CTAAGAACCTTTGTATTCCTTGATGTCTCGGATAACGCATTCCACGGAAGCATCCCTAAA 1020**

**LOC_Os01g06750_Nipponbare TCTATGGGAGAGCTAGTTCTGCTACATACGCTCAACATGTCACACAACTCACTGACAGGA 1080**

**LOC_Os01g06750_Pusa1342 TCTATGGGAGAGCTAGTTCTGCTACATACGCTCAACATGTCACACAACTCACTGACAGGA 1080**

**LOC_Os01g06750_PB1121 TCTATGGGAGAGCTAGTTCTGCTACATACGCTCAACATGTCACACAACTCACTGACAGGA 1080**

**LOC_Os01g06750_Nipponbare CCAATTCCATCTCAACTCGGCCGTCTAAAGCAGATGGAGGCTTTGGACCTATCTTCAAAT 1140**

**LOC_Os01g06750_Pusa1342 CCAATTCCATCTCAACTCGGCCGTCTAAAGCAGATGGAGGCTTTGGACCTATCTTCAAAT 1140**

**LOC_Os01g06750_PB1121 CCAATTCCATCTCAACTCGGCCGTCTAAAGCAGATGGAGGCTTTGGACCTATCTTCAAAT 1140**

**LOC_Os01g06750_Nipponbare GAGCTTTCAGGTGTCATTCCACAGGAATTACCATCCTTGGACTTCCTCGGAATGCTAAAT 1200**

**LOC_Os01g06750_Pusa1342 GAGCTTTCAGGTGTCATTCCACAGGAATTACCATCCTTGGACTTCCTCGGAATGCTAAAT 1200**

**LOC_Os01g06750_PB1121 GAGCTTTCAGGTGTCATTCCACAGGAATTACCATCCTTGGACTTCCTCGGAATGCTAAAT 1200**

**LOC_Os01g06750_Nipponbare CTATCCTACAACAGGCTGGAGGGGAAAATACCAGAATCGCTTCATTTTTCGTTATTCGCC 1260**

**LOC_Os01g06750_Pusa1342 CTATCCTACAACAGGCTGGAGGGGAAAATACCAGAATCGCTTCATTTTTCGTTATTCGCC 1260**

**LOC_Os01g06750_PB1121 CTATCCTACAACAGGCTGGAGGGGAAAATACCAGAATCGCTTCATTTTTCGTTATTCGCC 1260**

**LOC_Os01g06750_Nipponbare AATAGTTCATTTCTTGGGAATGATGCTTTATGTGGCCCTCCTCTATCTAAAGGCTGCAGC 1320**

**LOC_Os01g06750_Pusa1342 AATAGTTCATTTCTTGGGAATGATGCTTTATGTGGCCCTCCTCTATCTAAAGGCTGCAGC 1320**

**LOC_Os01g06750_PB1121 AATAGTTCATTTCTTGGGAATGATGCTTTATGTGGCCCTCCTCTATCTAAAGGCTGCAGC 1320**

**LOC_Os01g06750_Nipponbare AACATGACATTGCCGAATGTGATACCTTCCGAGAAGAAATCTGTAGATGTTATGCTGTTC 1380**

**LOC_Os01g06750_Pusa1342 AACATGACATTGCCGAATGTGATACCTTCCGAGAAGAAATCTGTAGATGTTATGCTGTTC 1380**

**LOC_Os01g06750_PB1121 AACATGACATTGCCGAATGTGATACCTTCCGAGAAGAAATCTGTAGATGTTATGCTGTTC 1380**

**LOC_Os01g06750_Nipponbare CTCTTTTCTGGAATAGGATTTGGCCTTGGATTTGCCATTGCAATTGTAGGAGCATGGGGA 1440**

**LOC_Os01g06750_Pusa1342 CTCTTTTCTGGAATAGGATTTGGCCTTGGATTTGCCATTGCAATTGTAGGAGCATGGGGA 1440**

**LOC_Os01g06750_PB1121 CTCTTTTCTGGAATAGGATTTGGCCTTGGATTTGCCATTGCAATTGTAGGAGCATGGGGA 1440**

**LOC_Os01g06750_Nipponbare ATTCCCATTAGAAGACGGTCTCCGGCGAGGCAGAGAGCCCTCTGA 1485**

**LOC_Os01g06750_Pusa1342 ATTCCCATTAGAAGACGGTCTCCGGCGAGGCAGAGAGCCCTCTGA 1485**

**LOC_Os01g06750_PB1121 ATTCCCATTAGAAGACGGTCTCCGGCGAGGCAGAGAGCCCTCTGA 1485**

***LOC_Os01g06870***

**LOC_Os01g06870_Nipponbare ATGATGATATCCACCAAGCAACGCCTCCTCACTCCCATTCTCATTCTCCTCATTTGTTGT 60**

**LOC_Os01g06870_Pusa1342 ATGATGATATCCACCAAGCAACGCCTCCTCACTCCCATTCTCATTCTCCTCATTTGTTGT 60**

**LOC_Os01g06870_PB1121 ATGATGATATCCACCAAGCAACGCCTCCTCACTCCCATTCTCATTCTCCTCATTTGTTGT 60**

**LOC_Os01g06870_Nipponbare TACTCCATAGTCACTGCAGCCAACAATGACACAACCGTCCCTTGTCTCCCAGAGCAAGCT 120**

**LOC_Os01g06870_Pusa1342 TACTCCATAGTCACTGCAGCCAACAATGACACAACCGTCCCTTGTCTCCCAGAGCAAGCT 120**

**LOC_Os01g06870_PB1121 TACTCCATAGTCACTGCAGCCAACAATGACACAACCGTCCCTTGTCTCCCAGAGCAAGCT 120**

**LOC_Os01g06870_Nipponbare TCTTCCCTCCTACAGCTCAAAAATTCCTTCATCAATAATGCAAACTTATCGTCATGGCGA 180**

**LOC_Os01g06870_Pusa1342 TCTTCCCTCCTACAGCTCAAAAATTCCTTCATCAATAATGCAAACCTATCGTCATGGCGA 180**

**LOC_Os01g06870_PB1121 TCTTCCCTCCTACAGCTCAAAAATTCCTTCATCAATAATGCAAACTTATCGTCATGGCGA 180**

**LOC_Os01g06870_Nipponbare GCTGGATCGGACTGCTGCCACTGGGAGGGCATCACATGTGGTATGGCCTCCGGCAGGGTC 240**

**LOC_Os01g06870_Pusa1342 GCTGGATCGGACTGCTGCCACTGGGAGGGCATCACCTGTGGTATGGCCTCCGGCAGGGTC 240**

**LOC_Os01g06870_PB1121 GCTGGATCGGACTGCTGCCACTGGGAGGGCATCACATGTGGTATGGCCTCCGGCAGGGTC 240**

**LOC_Os01g06870_Nipponbare ATTTCCCTTGACCTCAGTGAGTTGAACTTGATGAGCAATCGTCTCGATGCTGCACTCTTT 300**

**LOC_Os01g06870_Pusa1342 ATTTCCCTTGACCTCAGTGGGTTGAACTTGATGAGCAATCGTCTCGATGCTGCACTCTTT 300**

**LOC_Os01g06870_PB1121 ATTTCCCTTGACCTCAGTGAGTTGAACTTGATGAGCAATCGTCTCGATGCTGCACTCTTT 300**

**LOC_Os01g06870_Nipponbare AACCTCACCTCTCTTACAAACCTTAACCTTGCTTCCAATTACTTTTGGAGAGCCGAACTC 360**

**LOC_Os01g06870_Pusa1342 AACCTCACCTCTCTTAGAAACCTTAACCTTGCTTCCAATTACTTTGGGAAAGCCCCACTC 360**

**LOC_Os01g06870_PB1121 AACCTCACCTCTCTTACAAACCTTAACCTTGCTTCCAATTACTTTTGGAGAGCCGAACTC 360**

**LOC_Os01g06870_Nipponbare CCAGTATCTGGGTTTGAGAGGCTCACAGATATGATCCACCTCAACTTCTCACACAGCAAC 420**

**LOC_Os01g06870_Pusa1342 CCAGTTTCTGGGTTTGAGAGGCTCACAGATATGATCGACCTCAACTTCTCCCACAGCAAC 420**

**LOC_Os01g06870_PB1121 CCAGTATCTGGGTTTGAGAGGCTCACAGATATGATCCACCTCAACTTCTCACACAGCAAC 420**

**LOC_Os01g06870_Nipponbare TTTTATGGTCAGATCCCCATTGGACTTGCTTGCCTCATGAAACTTGTCACTCTTGACTTT 480**

**LOC_Os01g06870_Pusa1342 TTTTATGGTCAGATCCCCATTGGACTTGCTTGCCTCATGAAACTTGTCACTCTTGACTTT 480**

**LOC_Os01g06870_PB1121 TTTTATGGTCAGATCCCCATTGGACTTGCTTGCCTCATGAAACTTGTCACTCTTGACTTT 480**

**LOC_Os01g06870_Nipponbare TCTAGTAATGATGGGTTGTATTTCGATGAACCAAGTTTCCAAACCGTCATGGCAAACATG 540**

**LOC_Os01g06870_Pusa1342 TCTAGTAATTATGGGTTGTATTTCGATGAACCAAGTTTCCAAACCGTCATGGCAAACATG 540**

**LOC_Os01g06870_PB1121 TCTAGTAATGATGGGTTGTATTTCGATGAACCAAGTTTCCAAACCGTCATGGCAAACATG 540**

**LOC_Os01g06870_Nipponbare AGCAACCTGAGGGAGCTCCATCTTGATGAGATAGAAATTTTCGGATCAACTTGGTCAGTT 600**

**LOC_Os01g06870_Pusa1342 AGCAACCTGAGGGAGCTCCATCTTGATGAGATAGAAATTTTCGGATCAACTTGGTCAGCT 600**

**LOC_Os01g06870_PB1121 AGCAACCTGAGGGAGCTCCATCTTGATGAGATAGAAATTTTCGGATCAACTTGGTCAGTT 600**

**LOC_Os01g06870_Nipponbare GTTTTAGCAGACAACATTCCTCAACTCGAGATTCTTAGCTTGTTTGCGTGCCGTATATCT 660**

**LOC_Os01g06870_Pusa1342 GTTTTAGCAGACAACATTCCTCAACTCGAGATTCTTAGCTTGCTTGCGTGCCGTATATCA 660**

**LOC_Os01g06870_PB1121 GTTTTAGCAGACAACATTCCTCAACTCGAGATTCTTAGCTTGTTTGCGTGCCGTATATCT 660**

**LOC_Os01g06870_Nipponbare GGTCCTATTCACTCTTCCTTCTCAAGGCTTCGTTCCTTAAAGGTGATCAACCTTGGATAC 720**

**LOC_Os01g06870_Pusa1342 GGTCCTATTCACTCTTCATTCTCAAGGCTTCGTTCCTTAAAGGTGATCAACCTTGGATAC 720**

**LOC_Os01g06870_PB1121 GGTCCTATTCACTCTTCCTTCTCAAGGCTTCGTTCCTTAAAGGTGATCAACCTTGGATAC 720**

**LOC_Os01g06870_Nipponbare AATTTTGGACTCCCCAGCAAAGTTCCTGAGTTCTGTGCTGAATTATCTTCTTTGAGCATT 780**

**LOC_Os01g06870_Pusa1342 AGTTTTGAACTCCCCAGCAAAGTTCCTGAGTTCTTTGCTGAATTATCTTCTTTGAGCATT 780**

**LOC_Os01g06870_PB1121 AATTTTGGACTCCCCAGCAAAGTTCCTGAGTTCTGTGCTGAATTATCTTCTTTGAGCATT 780**

**LOC_Os01g06870_Nipponbare CTTGAAATTGCAGGCAATTCTTTTGAAGGGCAGTTCCCAACAAAAATCTTCCACCTAAAA 840**

**LOC_Os01g06870_Pusa1342 CTTGAAATTGCAGGCAATTCTTTTGAAGGGCAGTTCCCAACAAAAATCTTCCACCTAAAA 840**

**LOC_Os01g06870_PB1121 CTTGAAATTGCAGGCAATTCTTTTGAAGGGCAGTTCCCAACAAAAATCTTCCACCTAAAA 840**

**LOC_Os01g06870_Nipponbare AGTTTGAGAACGCTTGATTTGTCTCATAACCCCAATCTTTCTATTAACCTACCAGAATTC 900**

**LOC_Os01g06870_Pusa1342 AGATTGAGAACGCTTGATTTGTCTCATAACCCCAATCTTTCTATTAACCTACCAGAATTC 900**

**LOC_Os01g06870_PB1121 AGTTTGAGAACGCTTGATTTGTCTCATAACCCCAATCTTTCTATTAACCTACCAGAATTC 900**

**LOC_Os01g06870_Nipponbare CCTGATGGAAATAATCTAGAAACACTAGGTCTAGCAGCAACCAATTTATCTTATCACATA 960**

**LOC_Os01g06870_Pusa1342 CCTGATGGAAATAATCTAGAAACACTAGATCTAGCAGCAACCAATTTATCTTATCACATA 960**

**LOC_Os01g06870_PB1121 CCTGATGGAAATAATCTAGAAACACTAGGTCTAGCAGCAACCAATTTATCTTATCACATA 960**

**LOC_Os01g06870_Nipponbare CCGTCCTCATTTGCCAATCTCAAGTCCTTGAAGCGTTTGGGTATGAGCACGGCACGAACT 1020**

**LOC_Os01g06870_Pusa1342 CCGTCCTCATTTGCCAATCTCAAGTCCTTGAAGCGTTTGAGTATGAGCACGGCACGAATT 1020**

**LOC_Os01g06870_PB1121 CCGTCCTCATTTGCCAATCTCAAGTCCTTGAAGCGTTTGGGTATGAGCACGGCACGAACT 1020**

**LOC_Os01g06870_Nipponbare TCCAAGGAGCTGCCCTCTTTATTAGATAAGCTTCCTTCATTGACAGAACTGGAATTGCAA 1080**

**LOC_Os01g06870_Pusa1342 TCCAAGGAGCTGCCCTGTTTATTAGGTAAGCTTCCTTCATTGACAAAACTGGAATTGCAA 1080**

**LOC_Os01g06870_PB1121 TCCAAGGAGCTGCCCTCTTTATTAGATAAGCTTCCTTCATTGACAGAACTGGAATTGCAA 1080**

**LOC_Os01g06870_Nipponbare GGATCGGAATCAGGCTTGGAGAAGGCGGTATTATCTTGGGTAGGCAACCTTAAGCAGCTG 1140**

**LOC_Os01g06870_Pusa1342 GGATCGGAATCAGGCTTGGAGAAGGCGGTATTATCTTGGGTAGGCAACCTTAAGCAGCTG 1140**

**LOC_Os01g06870_PB1121 GGATCGGAATCAGGCTTGGAGAAGGCGGTATTATCTTGGGTAGGCAACCTTAAGCAGCTG 1140**

**LOC_Os01g06870_Nipponbare ACTGCTTTGGAGCTTGTATCGTACGATTTCTCTGAATCGGCACCCTCCTGGATAGGCAAT 1200**

**LOC_Os01g06870_Pusa1342 ACTGCTTTGGAGCTTGTATCGTACGATTTCTCTGAATCGGCACCCTCCTGGATAGGCAAT 1200**

**LOC_Os01g06870_PB1121 ACTGCTTTGGAGCTTGTATCGTACGATTTCTCTGAATCGGCACCCTCCTGGATAGGCAAT 1200**

**LOC_Os01g06870_Nipponbare TTGACGAATTTGAAGTTTTTGTGGATCTGGGACTGCAATTTCTCTGGGTCAATAATACCA 1260**

**LOC_Os01g06870_Pusa1342 TTGACGAATCTGAAGTTTTTGTGGATCTGGGACTGCAATTTCTCTGGGTCAATAATACCA 1260**

**LOC_Os01g06870_PB1121 TTGACGAATTTGAAGTTTTTGTGGATCTGGGACTGCAATTTCTCTGGGTCAATAATACCA 1260**

**LOC_Os01g06870_Nipponbare TATCAGATTGGCAATCTTGCAAAATTGGAGACCTTGGACTTTAGAGGGTGCGAGTTCTTT 1320**

**LOC_Os01g06870_Pusa1342 TATCAGATTGGCAATCTTGCAAAATTGGAGACCTTGGACTTTAGAGGGTGCGAGTTCTTT 1320**

**LOC_Os01g06870_PB1121 TATCAGATTGGCAATCTTGCAAAATTGGAGACCTTGGACTTTAGAGGGTGCGAGTTCTTT 1320**

**LOC_Os01g06870_Nipponbare GGACAGCAAATACCACCGTGGATCGGTAATTTTACGAAGTTGGCAAATTTAGAGATGGAT 1380**

**LOC_Os01g06870_Pusa1342 GGACAGCAAATACCACCGTGGATTGGTAATTTTACGAAGTTGGCAAATTTAGAGATGGAT 1380**

**LOC_Os01g06870_PB1121 GGACAGCAAATACCACCGTGGATCGGTAATTTTACGAAGTTGGCAAATTTAGAGATGGAT 1380**

**LOC_Os01g06870_Nipponbare AGTTGTGGCTTCTCTGGGTCGATACCCTCAACAATAGGGAATCTGACCCAACTGGAAAGT 1440**

**LOC_Os01g06870_Pusa1342 AGTTGTGGCTTCTCTGGGTCGATACCCTCAACAATAGGGAATCTGACCCAACTGGGAAGT 1440**

**LOC_Os01g06870_PB1121 AGTTGTGGCTTCTCTGGATCGATACCCTCAACAATAGGGAATCTGACCCAACTGGAAAGT 1440**

**LOC_Os01g06870_Nipponbare CTGCGGATCACATCTAATCCCCAACTCAACGGTAAGTCTTTATACGACGGCTACTTCTTC 1500**

**LOC_Os01g06870_Pusa1342 CTGCGGATCACATCTAATCCCCAACTCAACGGTAAGTCTTTATACCACGGCTACTTCTTC 1500**

**LOC_Os01g06870_PB1121 CTGCGGATCACATCTAATCCCCAACTCAACGGTAAGTCTTTATACGACGGCTACTTCTTC 1500**

**LOC_Os01g06870_Nipponbare CTTCATACATATGCAATTTAA 1521**

**LOC_Os01g06870_Pusa1342 CTTCATACATATGCAATTTAA 1521**

**LOC_Os01g06870_PB1121 CTTCATACATATGCAATTTAA 1521**
