# Supplementary figures and images for: Fine mapping of qBK1.2, a major QTL governing resistance to bakanae disease in rice
Source: Front Plant Sci. 2023 Nov 10;14:1265176. doi: 10.3389/fpls.2023.1265176 (PMC10667430; doi:10.3389/fpls.2023.1265176)

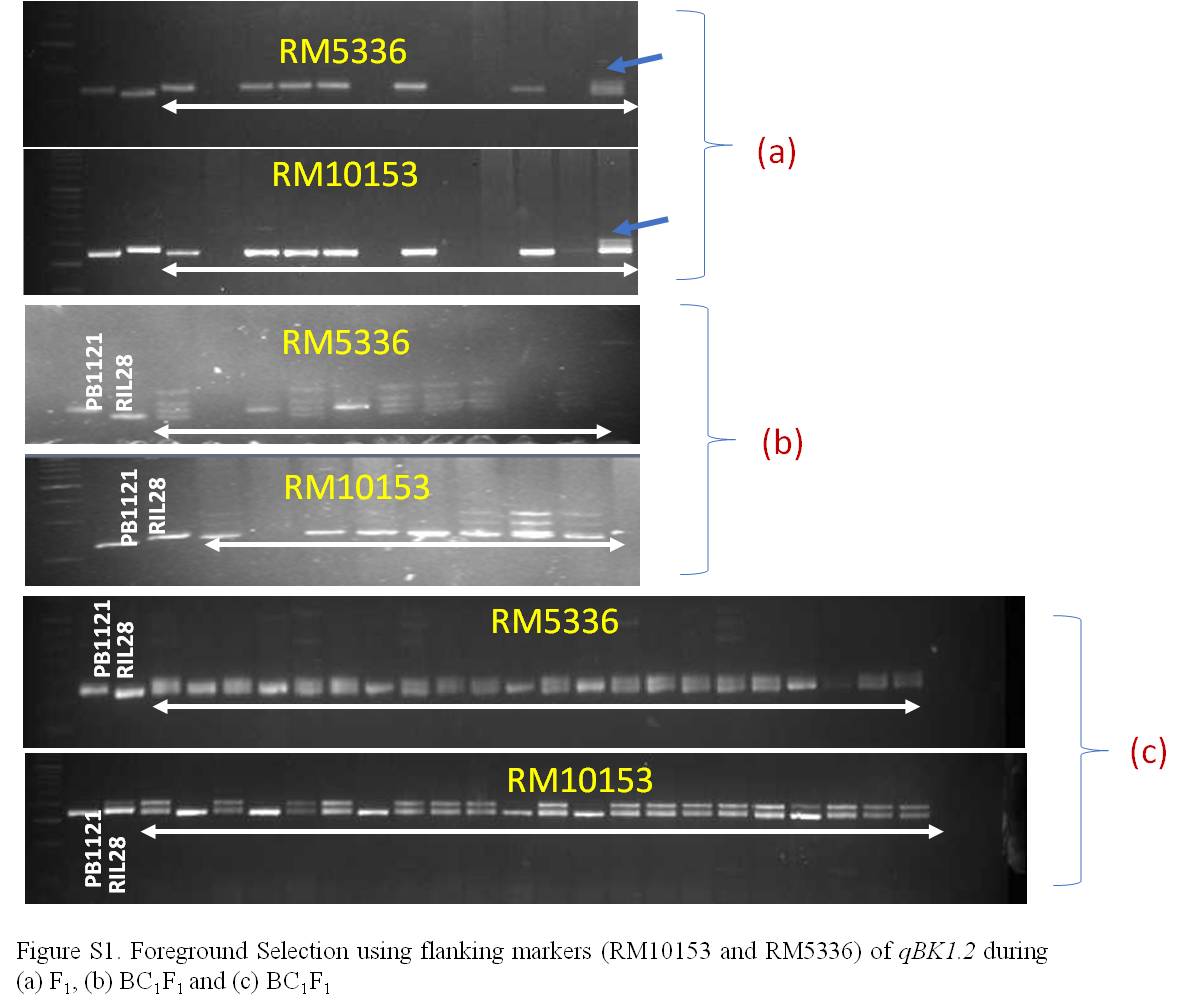

Supplement: Supplementary file 2 [file Image_1.jpeg]

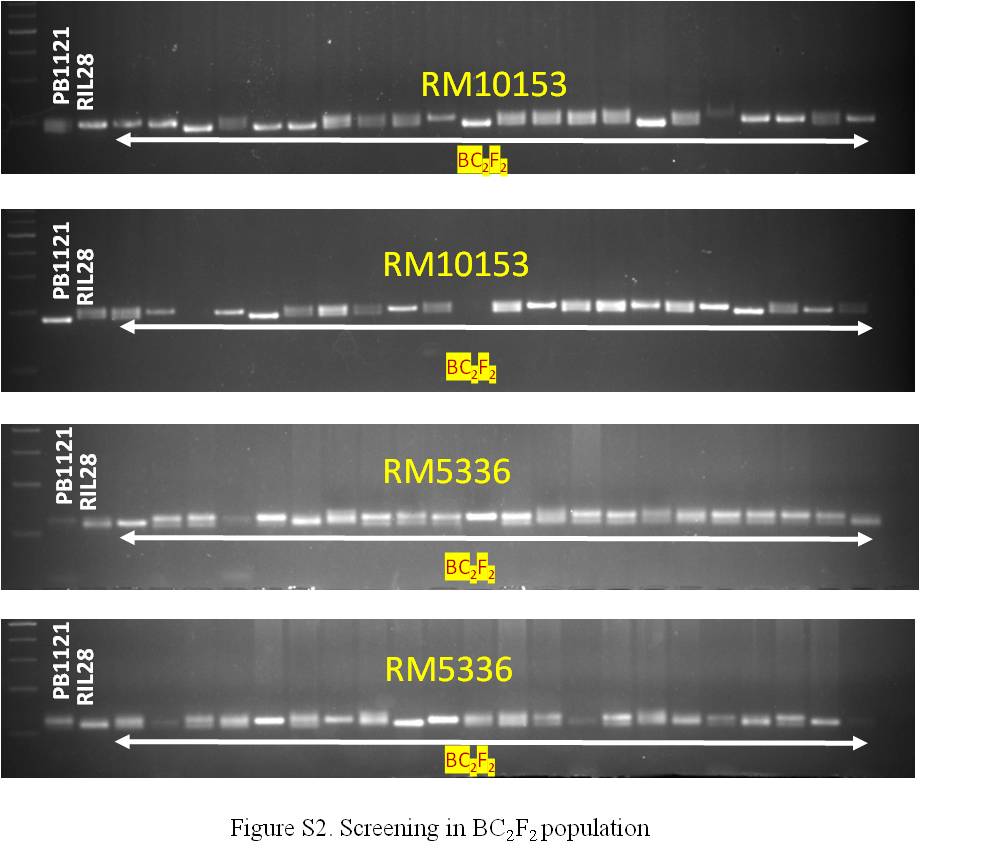

Supplement: Supplementary file 3 [file Image_2.jpeg]
