# Supplementary material for: Fine mapping of qBK1.2, a major QTL governing resistance to bakanae disease in rice
Source: Front Plant Sci. 2023 Nov 10;14:1265176. doi: 10.3389/fpls.2023.1265176 (PMC10667430; doi:10.3389/fpls.2023.1265176)
Supplement: Supplementary file 4 [file Table_1.docx]

**Supplementary Table 1. List of polymorphic markers between Pusa Basmati 1121 and RIL28**

| **Marker** | **Forward** | **Reverse** |
| --- | --- | --- |
| RM1282 | TCGTGCAGGAGGTCTTCATGG | TTGAGGATGGTAACGAACCTTGC |
| RM8069 | CGTTCAAAGCGAGCTTAATTGC | CTACGGCGGCTAAACATAACTCC |
| RM10217 | GCACTCACTCTCACTGATCACTTGC | CTCGTGTTGTGTTGGTGTGTTGC |
| RM578 | AGATATACACGGCAATCCGATCC | GTAGGGTTTGAAAGCTTGAAGTGC |
| RM493 | GTACGTAAACGCGGAAGGTGACG | CGACGTACGAGATGCCGATCC |
| RM572 | CGGTTAATGTCATCTGATTGG | TTCGAGATCCAAGACTGACC |
| RM11597 | GTATTTATTGTGGGCGGCAAACC | CGTTTGCTAGTTCAGTGTTGTGTCG |
| RM1152 | TGCTCTTATACCGTTCACATCC | TCAAACTAACCCTTCTGAGAGC |
| RM3646 | GACACCGAGGTCCACAAGAGG | CTTGACACGGCAAGGCTATCG |
| RM520 | ACGATAACGCCGACATCACTGG | GCTAAGCATCCACGGTTTCTCTCC |
| RM16388 | CGGAGAAGATTAGCAGTGATGG | GCACGTACTACTCCATCTGTTTCG |
| RM7200 | GCTGCACTATGCAGTTGCTGAGG | TGATCGATGGTGACGATGATACGC |
| RM471 | AGAAATGGATCGGACTGAACATGC | AGACACTCGGACGCACAAGC |
| RM127 | CGAAGCTTTCGGTGGGATAGC | ACCTTGAGCGAGTCCTTGAACG |
| RM7639 | GGTAGGCACCCACCCACATTGC | GTCGTTCTCCGCCACCTTCTCC |
| RM164 | TCTTGCCCGTCACTGCAGATATCC | GCAGCCCTAATGCTACAATTCTTC |
| RM440 | GGTAGGCACCAAAGAGTTTGACG | GGCATCACCTTATCCAATCACC |
| RM7639 | GGTAGGCACCCACCCACATTGC | GTCGTTCTCCGCCACCTTCTCC |
| RM6458 | TTGTCACGAGAGATGTGAGAGTGAGC | GGGTCTTCGAGGATGGAGTTGG |
| RM152 | GAAACCACCACACCTCACCG | CCGTAGACCTTCTTGAAGTAG |
| RM7390 | TGAGAGCTCGTAGGAAGTGTCC | CAGAGTCAGCAATCGCTAAGG |
| RM185 | GGCTCTCCATCTCCATTGATCC | GAGTTGTTGGGAGGGAGAAAGG |
| RM552 | CGCAGTTGTGGATTTCAGTGC | TCATGCTCAACGTTTGACTGTCC |
| RM3103 | CTGGAGTGGAGAAGAGAGAACAGG | TCTCCGCTCGGTTTCATCTAGG |
| RM28090 | ATCGATCCTCAAGGCAGCATGG | GGCTTGGAAGTTCAGGCACAGG |
| RM1261 | ATGGTAGAGACACAAGTCCATGC | GACAAATTGGTGTAGGTGAAGG |
| RM277 | CGGTCAAATCATCACCTGAC | CAAGGCTTGCAAGGGAAG |
| RM1246 | GGCTCACCTCGTTCTCGATCC | CATAAATAAATAGGGCGCCACACC |
