# Supplementary material for: Fine mapping of qBK1.2, a major QTL governing resistance to bakanae disease in rice
Source: Front Plant Sci. 2023 Nov 10;14:1265176. doi: 10.3389/fpls.2023.1265176 (PMC10667430; doi:10.3389/fpls.2023.1265176)
Supplement: Supplementary file 5 [file Table_2.docx]

**Supplementary Table 2. Marker used for foreground selection**

| **Marker** | **Forward** | **Reverse** |
| --- | --- | --- |
| RM10153 | GCGACCGAATAAATTTCCAAGG | AAATACGAGAGCCCTTTCCATCC |
| RM5336 | ACCCATCACCATGGCATCAATCC | GATCCCGCGGGTTTCGTAGC |
