# Supplementary material for: Fine mapping of qBK1.2, a major QTL governing resistance to bakanae disease in rice
Source: Front Plant Sci. 2023 Nov 10;14:1265176. doi: 10.3389/fpls.2023.1265176 (PMC10667430; doi:10.3389/fpls.2023.1265176)
Supplement: Supplementary file 6 [file Table_3.docx]

**Supplementary Table 3. List of candidate gene within fine mapped region**

| **Gene ID** | **Putative Function (RAP-DB annotation)** |
| --- | --- |
| **LOC_Os01g06720** | **Disease resistance protein SlVe2 precursor, putative, expressed** |
| **LOC_Os01g06730** | **Verticillium wilt disease resistance protein, putative, expressed** |
| LOC_Os01g06740 | Ribosome inactivating protein, putatively, expressed |
| **LOC_Os01g06750** | **Verticillium wilt disease resistance protein precursor, expressed** |
| **LOC_Os01g06760** | **Verticillium wilt disease resistance protein Ve2, putative, expressed** |
| LOC_Os01g06770 | Retrotranposon protein, putative, Ty3-gypsy subclass, expressed |
| LOC_Os01g06780 | Retrotranposon protein, putative, Ty3-gypsy subclass |
| **LOC_Os01g06790** | **Disease resistance protein, putative, expressed** |
| LOC_Os01g06800 | Expressed protein |
| LOC_Os01g06810 | Retrotranposon protein, putative, Ty1-copia subclass expressed |
| LOC_Os01g06820 | Hcr2-0B, putative, expressed |
| **LOC_Os01g06836** | **Disease resistance protein SlVe2 precursor, putative, expressed** |
| LOC_Os01g06852 | Transposon protein, putative, unclassified, expressed |
| **LOC_Os01g06870** | **Resistance protein SlVe1 precursor, putative, expressed** |
| **LOC_Os01g06876** | **Cf-2, putative, expressed** |
| LOC_Os01g06882 | Expressed protein |
| **LOC_Os01g06890** | **Leucine-rich repeat family protein, putative, expressed** |
| **LOC_Os01g06900** | **Verticillium wilt disease resistance protein Ve2, putative, expressed** |
| LOC_Os01g06910 | Expressed protein |
| **LOC_Os01g06920** | **Resistance protein SlVe1 precursor, putative, expressed** |
| LOC_Os01g06940 | POEI33-Pollen Ole e I allergen and extensin family protein precursor expressed |
